# The empirical study of norms is just what we are missing

**DOI:** 10.3389/fpsyg.2014.01159

**Published:** 2014-10-20

**Authors:** Theodora Achourioti, Andrew J. B. Fugard, Keith Stenning

**Affiliations:** ^1^Institute for Logic, Language and Computation, University of AmsterdamAmsterdam, Netherlands; ^2^Department of Clinical, Educational and Health Psychology, University College LondonLondon, UK; ^3^Department of Psychology, University of GießenGiessen, Germany

**Keywords:** reasoning goals, normativity, reasoning norms, syllogisms, classical logic, nonmonotonic reasoning, probabilistic reasoning, heterogeneity of human reasoning

## Abstract

This paper argues that the goals people have when reasoning determine their own norms of reasoning. A radical descriptivism which avoids norms never worked for any science; nor can it work for the psychology of reasoning. Norms as we understand them are illustrated with examples from categorical syllogistic reasoning and the “new paradigm” of subjective probabilities. We argue that many formal systems are required for psychology: classical logic, non-monotonic logics, probability logics, relevance logic, and others. One of the hardest challenges is working out what goals reasoners have and choosing and tailoring the appropriate logics to model the norms those goals imply.

## 1. Introduction

Formal systems offer a precise way to characterize people's various reasoning goals. There are many logics for different situations. Some allow reasoners to withdraw conclusions as more information is learned. Others describe the logic of deontic rules about “ought” and “must.” There are logics for relevance and for probabilities. Each logic provides different norms, e.g., for what constitutes a valid logical argument or whether a sentence is true. Elqayam and Evans (2011) propose that normativity in psychological practice should be avoided. We cannot see how. In this article we argue that without norms of some kind, we cannot interpret the data participants produce. Rather, participants' reasoning goals generate their own norms of reasoning and logics provide a good way to capture these norms. Pure descriptivism is impossible, and highly undesirable.

We first remind the reader of the distinction between *constitutive* and *regulative* norms which plays an important role in this paper. Constitutive norms define a certain behavior for what it is (see Searle, 1969). Characteristic examples are the rules of a game, e.g., the game of chess: changing the rules means playing a different game. Norms are regulative rather than constitutive when they do not define but regulate a preexisting activity. In this sense, regulative norms are not necessary and they are also derivative: they are consequences of constitutive norms, together with contextual features such as overall goals or specific constraints. For instance, what move to perform at any point when playing a game of chess is dictated by regulative norms: it may be that one wants to lose and terminate the game as soon as possible. Even with this unusual contextual goal, the revised regulative norms arise from the usual constitutive norms. Importantly, regulative norms are action oriented, in the sense that they tell one what to do.

Formal systems are instrumental in specifying constitutive and regulative norms, which is in turn necessary in order to understand what participants do in a particular reasoning task. Formal systems are characterized by constitutive norms: doing arithmetic is constituted by complying with the well known constitutive norms of arithmetic. And constitutive norms give rise to regulative norms (Achourioti et al., 2011). If you are dealing with numbers that represent prices of items, and you want a total, then adding them is permissible—a regulative norm. If you are dealing with numbers which are barcode identifiers and you want to count tokens (stocktaking perhaps?), then adding two of them is nonsense—another regulative norm. Formal systems impose regulative norms on non-formal activities that use them, and they do it as a consequence of their constitutive norms. Not uniquely of course, as our examples of trying to lose at chess, and different activities with numbers show. What the regulative norm is depends on the goals and other contextual features at hand; and as goals may be radically different (think of our earlier example of someone playing chess to lose), the regulative norms they generate may be radically different too.

Norms and values are, in the crucial cases for the psychology of reasoning, the least observable features of thinking—the farthest from being fixed by data without system or theory. Participants generally cannot describe their goals in the terms of appropriate systems or theory. Their performances nevertheless can provide evidence for theory-relative normative specification of goals, once a formal analysis is available. In this paper we illustrate these points with experimental examples.

There certainly are abuses of norms to be observed. We propose that these are most evident when any single homogeneous system account of human reasoning is proposed, whether it be classical logic (CL), probability theory, or indeed radical descriptivism with a single description language. As soon as hegemony is proposed, it becomes impossible to study the basis for selection from among multiple systems of reasoning, and it is this requirement to select from multiple possible systems that most clearly dissolves perceived problems of normativity, and connects reasoning goals to instrumental goals. Selecting from multiple possible reasoning goals can be done on instrumental grounds suiting the goals to the problem at hand. We do not believe there is any such thing as “human reasoning” construed as a homogenous system for the simple reason that the demands of different reasoning problems are incompatible, as we illustrate below. The main reasoning goal of this paper it to illustrate this point with examples from past and current practice.

The backdrop to our approach to norms and normativity is the multiple-logics approach to human reasoning outlined in Stenning and van Lambalgen (2008). It is widely accepted in modern logic that there are many logics which capture many kinds of reasoning, often incompatible one with another. They are best thought of as mathematical models of pure archetypes of reasoning. Logics have been around for a while, however, with notable exceptions, psychology still mostly uses only classical (“textbook” logic) and probability logics, and often rejects the idea that the latter even is a logic. What goes for logics goes more generally for formal systems used for modeling cognition. We therefore begin by providing some triangulation points better known to psychologists that relate this framework to possibly more familiar territory.

Todd et al. (2012) have proposed a multiple heuristics approach to decision making which makes the choice of alternative methods a contextualized choice, and in this shares important features with our multiple-systems approach to reasoning. The resulting norms are content-dependent as argued by Gigerenzer (2001). Bayesian models are often viewed as the established norm in decision, as well as more recently in reasoning. Todd et al. (2012) argue against the universality of a probabilistic norm. The heuristics proposed are specialized, and logics are at a somewhat different level of analysis, so not easy to compare, but nevertheless the two approaches are more closely related than may at first appear. Existing neural networks which implement the nonmonotonic logic we use, Logic Programming (LP) (Stenning and van Lambalgen, 2008, chapter 7), along with the internal generation of statistics of the networks' operation, can supply the theory-relative conditional frequency information that is required to select for these heuristics the content that they require in context. The networks also provide lists of defeaters—conditions that defeat conditional inferences and contribute to determining confidence in causal conditional reasoning (Cummins, 1995). This therefore offers a qualitative system of graded uncertainty in intensional reasoning which is a competitor to Bayesian methods in some contexts, through implementing the decision heuristics just mentioned.

Stich (1990) “The Fragmentation of Reason” and this author's work more generally on cognitive pluralism, is chiefly focussed on cases where different people (or peoples) have different norms of reasoning for some reason of individual or cultural preference or habit. We are focussed on cases in which participants' various goals call for different logics or systems of reasoning in different contexts. At least at first pass, on our account, everyone ought to conform to the constitutive norms of classical logic if their goals are, say, classical mathematical proof or the settlement of a certain kind of dispute. Everyone ought to conform to the norms of some nonmonotonic logic such as LP if their goal is to tell a story. Everyone ought to conform to the norms of deontic logic if they want to reason about permissions and obligations. And so on. So, our proposal is not relativistic in the usual sense. It is relativistic only in the sense that people's goals and therefore their norms are variable in different contexts. This does not diminish the interest of Stich's topic, nor of the two topics' relatedness. Widlok and Stenning (submitted) sketch how a multiple-logics approach bears on the recurrent anthropological debate about whether different cultures have different logics. Using nonmonotonic LP to analyse the Mambila's discourse of divination by spider, it concludes that cultures vary in the social circumstances in which they bring logics to bear, but that a working hypothesis should be that they evidence the same range of logics in the range of contexts they experience. Spider divination in context looks a whole lot less irrational through these eyes.

Clearly many authors have proposed many heterogeneities in reasoning, such as what is conventionally meant by the phrase “individual differences” in psychology, individual variation in how “good” some performance is. We are here concerned with a specific type of (in)homogeneity of formal system (e.g., classical logic, probability, nonmonotonic logic, …). Elqayam (2012) proposes grounded rationality—essentially the avowedly uncontroversial proposal that there is more to rational reasoning and action than the adoption of a formal system. There is more because people differ in their cognitive capacities, cognitive costs, mundane aims, and all the other variables of bounded rationality, and more. Elqayam (2012) appears to associate normativism with the adoption of a single formal standard of reasoning (usually either classical logic or probability in some form), and proposes “descriptivism” as an alternative that can preserve variety. So we agree there is more to rational action than logics or formal systems, and that adoption of a single system is a mistake. But we disagree that “descriptivism” can be conceived as an alternative to multiple-systems, and propose that the mundane limitations of grounded and bounded rationality interact with the unavoidable choice of reasoning system among the other systems that are also required. It is this interaction that provides great opportunity and power to the empirical investigation of reasoning and rationality. Description is of course important, but is always theory- and goal-relative. Since there are many theories and goals, there are many descriptions, and description itself cannot solve the inevitable choice of interpretation problem.

Bounded rationality is a proposal (which we applaud) that rational action has to be understood as governed by the intersection of many systematic constraints. To take one of Simon's examples (Simon, 1972), if working memory limitations are an important bound on a particular reasoning task, then a theory of working memory will be required to intersect with the cognitive implementation of whatever reasoning system is at work, in order to understand how contextual features (whether we have pencil and paper, whether we are expert in the domain, …) affect performance, and therefore what constitutes rational action for us in context. Countless social bounds are also sources of systematic constraint. Many relevant features of any particular situation may be entirely due to coincidence, but their operation is nevertheless to be understood in terms of several systematic theories. Totally unsystematic constraints are not comprehensible, by hypothesis. Thus bounded (or grounded) rationality requires multiple simultaneous systematic formal accounts of all the relevant constraints. With these systems come constitutive norms; and with those constitutive norms come regulative norms. The fact that we are not currently in a position to specify the many systematic constraints in general terms, and that we can make some progress with rather *ad hoc* accounts of say working memory, does not make a theory of bounded rationality able to dispense with these intersecting generalizations^1^. Boundedness does not make rationality *ad hoc*. The boundedness of working memory may or may not be there because we *ought* to be bounded in memory (though see, for example, Hertwig and Todd, 2005 and MacGregor, 1987 where advantages of boundedness are proposed) but it generates regulative norms such as: for an important reasoning task that clearly overloads your unaided working memory, it is not rational, other things being equal, not to have a pencil and paper to hand. Although we deliberately use examples of norms arising from individual reasoning because they are how experimental psychology generally meets up with normative considerations, it is not hard to see that the regulative norms arising from the constitutive norms of the formal elements can rapidly reach into any social, ethical or political activity people engage in.

As yet another orientation point, we recall that more than one logic may operate within an activity. Elsewhere we have proposed that an account of how at least some kinds of argument work, requires an account of how adversarial classical and cooperative nonmonotonic logics have to work together (Stenning, 2002, chapter 5, Stenning, 2012) to capture the interplay between cooperative and adversarial relations in argument. Mercier and Sperber (2011) propose that reasoning evolved for argumentation. These authors define reasoning with respect to explicitly aware processes, relegating unconscious processes to mere “inference.” On our account, accounting for argumentation that calls on both non monotonic and monotonic logics means bridging what Mercier and Sperber divide between inference and reasoning. One might propose that once cooperative discourse became possible, argumentation about its interpretation inevitably followed, for monitoring and repairing breakdowns in understanding. Argumentation is inconceivable without the existence of cooperative discourse. Elsewhere, we have criticized adaptationist attempts to try to read evolutionary accounts from informal descriptions of current function (Stenning and van Lambalgen, 2008, chapter 6). What is first required is a deeper description of the phenotype: and that requires empirical description of goals and norms.

The plan of this paper is that the first section discusses norms as we understand them, and how they are incompatible with any pure descriptivism. We will concentrate on how participants' very own reasoning goals create variety in *internal* norms which need to be captured in logics before any data of reasoning becomes interpretable, and draw out some consequences for empirical research. If normativity itself is not the problem, it is not without its abuses. We see the homogeneous application of formal systems as a major problem. Once only one system is allowed (whether it is Bayesianism, or classical logic, or whatever) then there is no way of assessing why a system is an appropriate choice for modeling an instance of reasoning. It cannot be an appropriate choice because it is no longer a choice. If there is heterogeneity (many logics or other competence models) then there have to be criteria of application, and indeed choice can be made on instrumental grounds—that is by a match between logical properties and reasoning goals, as we illustrate.

The second section takes the psychological study of categorical syllogistic reasoning as an example to illustrate these points. It argues that the descriptivism prevailing for the last half of the 20th century was exactly what led to a catastrophic inattention to the participants' reasoning goals. It describes the pervasive ambiguity of reasoning experiments for participants, most of whom adopt nonmonotonic reasoning goals where experimenters assumed classical logical ones. It spells out how the contrasting reasoning goals are constituted in the properties of these two logics.

The distinctive properties of classical logic give guidance for design of a context which should improve the chances that we see classical reasoning—in this case a context of dispute. Some results from an ongoing experimental program show how the properties of classical logic which make it suitable for a model of a certain kind of dispute or demonstration are presented as a first indication of the rewards of this kind of empirical program. It provides clear evidence that this context produces more classical reasoning than the conventional draw-a-conclusion task. And perhaps more importantly, it shows how participants have surprising implicit knowledge of some of the peculiarities of classical logic. Psychologically, our goal should be assessing peoples' implicit knowledge and its contextual expression i.e., their implicit logical concepts, rather than their scores on some fixed-context arbitrary task which engenders variable and unspecified goals.

The third section pursues similar themes in the example of probabilistic reasoning. The idea that Bayesianism, or even probability, provides a new homogeneous norm for human reasoning, and for rational action in general, has supplanted the same role that was previously assigned to classical logic in theories of rationality. But probability theory fails to provide reasoning goals at levels comparable to the examples of the previous section. What is argued for is an analogous differentiation of “probability logics” to apply to different reasoning goals, bridging to neighboring logics in a friendly welcoming manner.

Finally we end with some conclusions about the empirical programs that should follow from our arguments for a multiple-logics view of human reasoning, based on the differentiated reasoning goals that this multiplicity affords, together with some comments about the very different view of the relation between logic and psychology which emerges.

## 2. Explaining normativity

The experimental work discussed in the next two sections is intended to emphasis the role of normativity in the psychology of reasoning and should be read as such. It becomes for this reason important that we clarify what we mean by “normativity” and we will do this by reference to Elqayam and Evans (2011) which argues for descriptive as opposed to normative approaches and encapsulates our main focus. This article was followed by a series of commentaries some of which present views that are close to the points we make here. But we find that in many cases the picture is rather blurred and clarification of the key concepts is much needed so that points of agreement or disagreement can be identified and an essential discussion on the foundations of psychology of reasoning can get off the ground. Importantly, many of the arguments put forward against the use of normative frameworks depend on a specific understanding of “normativity,” which we would like to challenge^2^.

Logic is often said to be a normative system contrasted with descriptive frameworks that psychologists use. But a logical framework in itself is not descriptive or normative; it is the *use* of a logic that can be descriptive or normative, and even classical logic can serve as a descriptive tool in situations where people are found to reason classically. As we discuss later, such situations do not only arise in specialized contexts such as mathematical reasoning but may be found in research areas as prominent as syllogism tasks or natural language conditional statements. The interesting, indeed normative, question then is what are the circumstances, if there are any, that trigger classical reasoning, and make it appropriate in the situation: when is CL adopted by the participant as their norm for the task? We will discuss how classical logic, and especially those characteristics of it that distinguish it from other formal frameworks, provide cues as to where to look for the goals that may make it appropriate. The same goes for any other logic or formal system.

The role of normativity in questions such as the one just stated is clearly not of the evaluative kind. Contrast this with the following:

“A normative theory asks evaluative ‘ought’ questions: ‘What *ought* to be the good use of negation in language?’ A normative approach contains an element of evaluation, a sense of ‘goodness’ and ‘badness’, or ‘right’ and ‘wrong’, that is absent from a purely competence account. In short, normative theories are ‘ought’-type theories; computational theories are ‘is’-type theories. Note that the competence theories and performance theories are both descriptive—what they share is the *is*. ” (Elqayam and Evans, 2011), p.239

Here the term “normative” takes on almost ethical connotations. To be sure, such questions of prescriptive “goodness” and “badness” are at best outdated and in any case certainly irrelevant to the study of human reasoning. Not so, however, for “right” and “wrong” questions, as witnessed, for example, when participants report “errors” in their own reasoning and correct themselves in the process (we see an example later in how people reason about uncertain conditionals). There is nothing ethically objectionable or evaluative to supposing that humans are not perfect thinking machines and sometimes commit errors or refrain from driving their reasoning all the way to its utmost consequences^3^. and the notion of “error” makes little sense outside a normative framework that specifies what counts as “right” inferencing and what as “wrong.” The pertinent question is rather: how can we talk about “correctness,” or “right” and “wrong,” without falling into the same old trap as when psychologists considered classical logic to be the arbitrer of human rationality?

Most of the reluctance to engage seriously with normative considerations comes from an understanding of norms as “external” to one's reasoning, that is, as set by someone other than the participant herself (often researchers). Objections to normativity disappear as soon as attention shifts to norms that are constitutive of one's own reasoning, meaning that they help define reasoning for what it is^4^. We do not deny that norms ‘set by other people’ (social norms) are important. But if it is only such norms that are objectionable the debate has been ill-specified, and the objections to norms should be suitably diluted. A way to trace “internal” norms is to identify the goals that underlie and drive one's reasoning process. Goals are highly complex and not easy to specify as they stem from various sources. They are not observable and they interact with each other in complicated ways. In reasoning experiments, for example, the participant has to decide how to go about solving the task, which depends on the participant's interpretation of what is asked of her, which in turn depends on pragmatic goals influencing natural language processing of instructions, how much is underdetermined by the experimenter's design and so on. But whatever the underlying goals turn out to be, it has to be recognized that they heavily influence the type of reasoning participants engage in. In the next section we discuss concrete examples of how different goals trigger different reasoning processes, and we show this by varying the context in order to generate different types of reasoning (and thereby different reasoning norms) and study the effects of this variation on the experimental data.

With the understanding of normativity that we propose as “internal” and not “external” to reasoning, the discussion of human rationality can be set on new grounds. Consider the following:

What seems to set apart normative rationality from other types of rationality is the “ougthness” involved in normativism. Bounded rationality, for example, is not bounded because it “ought” to be so. Instead, there are just biological limits to how large brains can grow and how much information and how many computational algorithms they can store and execute.' (Elqayam and Evans, 2011), p. 236

As mentioned above, even this is contentious in the literature: there may be distinct advantages to limited systems, and there is much evidence that human brain-size is under selective pressure from both directions. But we accept that resource bounds are a fact. Resource constraints certainly influence the reasoning that participants engage in; this is one of the reasons that may render classical model theoretic thinking intractable and force naive participants to resort to nonmonotonic example construction through preferred models, that leads to more manageable computational processes. But notice that participants are switching reasoning subgoals, not attempting the same goal with a different tool. Such limitations are part of what a formal model helps represent. They lie, for example, at the heart of the difference between monotonic and nonmonotonic systems. And justifying one model rather than another is clear evidence of normative status, even if the norms in this case could not be otherwise because of resource bounds. Elqayam and Evans (2011) follow Evans and Over (1996) in setting apart “normative” rationality from “instrumental,” “bounded,” “ecological” and “evolutionary” rationality. The way we understand normativity, it is integral part of all of these four types of rationality. In fact, most of the present paper discusses norms that are part of so-called “instrumental rationality.” Hence, we take issue with remarks as the following:

‘Some researchers have proposed that we should adopt alternative normative systems such as those based on information, probability, or decision theory (Oaksford and Chater, 1991, 1998a,b, 2007), while others proposed that at least some forms of rationality need not necessarily require a normative system at all (e.g. Evans, 1993, 2002; Evans and Over, 1996; Gigerenzer and Selten, 2001). By this position, organisms are rational if they act in such a manner as to achieve personal goals, and such rationality need not involve any normative rule following.’ (Elqayam and Evans, 2011), p.234

The message here is that achieving personal goals need not involve normative rule following. It must be clear by now that we take reasoning goals to be intrinsically normative in that they play a big role in the choice of one reasoning mode rather than another (without claiming that some conscious decision-making process of selection takes place, or that they are necessarily constituted as such in “rules”). Pragmatic goals of relevance, for example, are essentially normative when in some contexts they exclude the interpretation of a natural language “or” as the classical logic disjunction, ∨. Just as with the selection task, examination has to reveal these hidden normative systems behind, for example, ecological rationality. Martignon and Krauss (2003) argue that Gigerenzer's heuristics require Bayesian methods for their population with content in context. And Martignon et al. (in preparation) give an account of this same process based on nonmonotonic logic. Ecological rationality is up to its ears in normativity.

We have so far proposed an understanding of normativity as applying to the use of formal systems rather than attaching to the systems themselves and as involving questions of correctness that do not have evaluative connotations but refer to norms which are internal to human reasoning and constitutive of it. To clarify these points even further, we now discuss the status of competence theories and the “is-ought” fallacy which normative approaches are said to commit. Here is an interesting quote:

‘… arbitrating between competing normative systems is both crucial and far from easy. This is where the difference between normative and competence theories becomes critical. Competence theories are descriptive and can hence be supported by descriptive evidence. In contrast, can one support normative theory with descriptive evidence? Can one infer the *ought* from the *is*?’ (p. 240)

We do not agree that competence theories can be supported by descriptive evidence without normative considerations. It is especially competence theories that have to see beyond the data in order to account for the discrepancy between theory and observation. And at the same time it is a truism that the further one moves away from observable data the more difficult it becomes to actually test the theory. So how is it possible at once to model competence and stay as close as possible to actual performance? Competence theories have constitutive norms, and these norms generate regulative norms once their reasoning is embedded in action. Our examples in the next sections show how the various constitutive norms participants adopt for syllogistic and probabilistic reasoning (competence theories) generate regulative norms once embedded in actual reasoning. A proper understanding of the data depends on the choice of logical norm.

Elqayam and Evans (2011) argue that much of the experimental cognitive research is liable to the “is-ought” fallacy (or naturalistic fallacy as it is often called by philosophers). However, in order for this transition from “is” to “ought” to make sense, “is” and “ought” must be clearly separated, and we show in this paper that descriptive and normative matters cannot be so neatly set apart. A purely descriptive approach is simply unattainable, since what the participants “do” already depends on the theoretical framework within which one performs the observation and this theoretical framework must take into account the reasoning goals at hand, the latter clearly creating normative demands. The dependence of description on formal theory is clearly seen when incompatible descriptions match the same data; when, as we discuss, for instance, the same answer to a reasoning task could be generated by reasoning processes that are as different as monotonic and nonmonotonic logics.

Interestingly, Elqayam and Evans (2011) take the “is-ought” fallacy to be especially triggered in cases where more than one theory matches the data, which then lends support to descriptive theories in their approach^5^. But we believe that it is precisely the need to select among equally matching theories that proves descriptivism to be impossible, on the one hand, and what saves the psychologist from the homogeneity trap, on the other. There we think, is the real danger when studying human reasoning without making explicit the norms and goals involved; namely, the idea that a single theory can play the role of setting the basis, descriptive or normative, over which to design and assess all experimental work.

Having to arbitrate between formal models is not in itself a problem we should want to eliminate, but it becomes such a problem if it means having to choose between theories that claim to explain human reasoning as a whole. This is where a multiple-logics approach as advocated here offers an improvement in the way formal models are used: in order to account for differences between participants' reasoning within a particular task, we ask ourselves how we can modify the task so that these differences become apparent. This we find the most interesting experimental challenge, which relies, however, on being open to different formalizations sensitive to participants' underlying norms and goals. Formalizing involves representation of reasoning norms (which are goal-sensitive) as much as empirical engagement. And here is where a single descriptive framework, even if that were possible, is bound to fail: it offers no way to account for pervasive participant differences flowing from different goals, if all one is allowed to do is to “describe” participants' micro-behavior.

## 3. The syllogism as illustration

### 3.1. Reasoning goals as norms embodied in formal systems

The earliest paper on the psychology of the syllogism by Störring (1908) does not address the relation between logic and psychology at all, but employing great logical and psychological insight gets on with describing a small number of participants' responses to syllogistic problems. It identifies Aristotle's *ekthesis* as a good guide to participants' reasoning processes. This itself is remarkable, coming so soon after the “divorce” of logic and psychology, and the establishment of the latter as experimental science. By mid-century, Wason (1968) argues strongly against the very idea that logic bears any useful relation to human reasoning, claiming to demonstrate this fact experimentally with Piaget's theory as his target.

It was a further half century before Wason's interpretation of his experiment was prominently challenged in psychology (Chater and Oaksford, 1999; Stenning and van Lambalgen, 2001; Evans, 2002; Stenning and van Lambalgen, 2004) (but see also Wetherick, 1970) by showing how it rested on the assumption that classical logic had to be the goal of participants' supposedly failed reasoning in Wason's Task, for any of his arguments for irrationality to succeed. But it behooves someone so vehement that logic contributes nothing to understanding human reasoning to perhaps find out what constitutes a logic. This simultaneous coupling of explicit denial of the relevance of classical logic, with its under-the-counter adoption as *the* criterion of correct reasoning, stems directly from an avoidance of the issue of participants' goals in reasoning, and this in turn is a direct result of the suppression of formal specifications of reasoning goals, in favor of a proposed descriptivism treating “human reasoning” as an activity with a homogeneous goal. Wherever descriptivism is espoused we find tacit appeal to homogenous normativism.

As we shall see in our example of the syllogism, it is a difficult experimental question to even specify what empirical evidence is required to distinguish between monotonic and nonmonotonic reasoning in the syllogistic fragment. It has been assumed that merely instructing different reasoning criteria is sufficient to discriminate. The empirical problems of discriminating these goals has been largely ignored or denied, and their neglect stems directly from conflict of this difficulty of observation with the descriptivism which we lament. Once a formal specification of an alternative interpretation of the task is available, it is possible to launch a genuine empirical exploration of what participants may be trying to do.

It is not difficult to see why a multiple-logics stance defuses accusations of prescriptive normativism. As soon as there is explicitly acknowledged plurality, then the need for specification of appropriateness conditions for the different logics is clear for all to see. Fortunately, multiplicity brings with it the materials for an answer. Why is classical logic a good model for adversarial reasoning such as the settlement of dispute? Well, it is bivalent, admitting no intermediate truth values. It is extensional, which means the relevant questions of meaning are easily identified, if not necessarily decided, in agreeing premises. It is truth functional, with similar consequences—no hidden meanings can obscure the connection intended by an intensional conditional. It reasons from identified premises with fixed interpretations. Wandering premises are not good for dispute resolution. But above all, its concept of validity requires the preservation of truth in conclusions from true premises under *all* assignments of truth values.

Why is Logic Programming a good logic for cooperative reasoning about the effect on our preferred model of knowledge rich interpretation of new information? Well, the knowledge-base of conditionals corresponds to the long term regularities in the environment, along with the numerous exceptions to these regularities. Working memory holds the representation of the current preferred model of the focal situation (the “closed world”). The closure of the world is made possible by the restriction of expression which allows the rapid settlement of whether a particular proposition can be derived from the large knowledge base. And so on. Even these partial descriptions of the differences between the logics are enough to explain for many contexts whether classical or a nonmonotonic logic is appropriate. The norm can be seen to be appropriate to the goal. It is when human reasoning is assumed to be logically homogeneous, lack of adequate justification is inevitable. For example, there is a pervasive though not universal view in the psychology of reasoning that monotonic and nonmonotonic logics are two ways of “doing the same thing,” where the nonmonotonic logic is seen as a poor man's approximation to classical logic. For example, Mental Models theory correctly asserts that to achieve classical reasoning, participants should consider all models of the premises in syllogistic reasoning. But when it is clear that they mostly actually only consider one model, this is considered a performance error (forgetfulness): not a symptom of nonmonotonic goals to identify a preferred model. This is accompanied by separate experimental demonstrations that participants *can* successfully search for counterexample models when explicitly instructed to do so, in a quite different task. This is taken as supporting that indeed the failure to look for them in solving syllogisms is a performance error. At no point is it questioned whether the participants' goal is different in these two tasks. Just because people can do counterexample reasoning sometimes, does not mean that this is always their goal.

The LP machinery may often operate below awareness; this does not mean that the participant who adopted the goal that it performs does not “have” the goals under which it operates. And plurality is absolutely required for other reasons. There is no way that any logic can provide a model of both dispute and exposition because the logical properties listed above are incompatible^6^.

From these arguments it follows that pure descriptivism is impossible in situations where both CL and LP are live options for participants' interpretation (most laboratory reasoning tasks) because choice of logic, and with it reasoning goals, is required for interpretation of the data. There is no alternative to seeking evidence for which goals the participant has adopted (usually inexplicitly). Merely varying the instructions is not an adequate tool for discovery.

### 3.2. Descriptivist approaches to the syllogism cannot discriminate these goals

There are 64 pairs of syllogistic premises which can be enumerated with their valid conclusions. There are a some logical glitches about exactly what ought to be listed as valid ^7^. The conventional task for studying “syllogistic reasoning” is defined by the goal of “getting these answers” to the question “What follows from these premises?” For example, if the premises are *All A are B. All B are C* then *All A are C* is a valid conclusion. So participants who answer with this conclusion score a point. This is OK as far as it goes as an denationalization, but if it is all we can offer, then it makes the syllogism an uninteresting pursuit for the researcher and participant alike. Who says these ones are valid? So it is generally further assumed by the experimenter that these right answers are given by classical logic—was not Aristotle, the author of the first logical theory of syllogisms, thereby the inventor of classical logic?—but pure descriptivism is already out the window. CL has constitutive norms, and with them its users and uses acquire regulative norms.

Troubles compound. These participants have been selected for not knowing explicitly what the syllogism, or classical logic, are. It is true that they know the natural language of the premises, and it is easy to suppose that this determines the reasoning goal. But it is the *discourse* that they have trouble understanding out of context. And they often complain about the bizarreness of the discourse in ways that make one think they in fact adopt a goal quite different to the one the experimenter stipulates. For example, given *Some A are B. Some C are B* they frequently complain that “it doesn't tell me whether the Bs are the same or different.” This complaint makes no sense if the premises are understood “classically.” Classically it is absolutely clear that they could be either the same or different unless the quantifiers force them to be related, and in this case they “obviously” do not. Yet about 60% of participants claim that there is a valid conclusion here^8^ On a “story-understanding” LP interpretation, they are of course right that the discourse is “defective” and there are ways of fixing it so that there are valid conclusions based on preferred models—several ways.

So we do not yet know what the participants' goals are at any level beyond assuming they are to please the experimenter, who has not been good enough to divulge his goals in a way that the participant can interpret them. Just saying “I want what logically follows” or “what must be true” is not helpful, since “logically” has many meanings in the vernacular (“reason carefully” is often a good gloss), and any participants who have taken intro logic have been weeded out. “Logically” also has many technical meanings. In LP, a conclusion *must* be true (in the current context) if it follows in the current context from the preferred model. The psychological effects of this kind of emphatic instruction are congruent with the idea that participants take a little more care with whatever goals they happen to have.

Why should we care? What clarification of the goals of the participants would make the syllogism more interesting? We should care about the syllogism because it is a suitable microcosm for seeking the psychological foundations of classical logical reasoning, if any, and that is interesting because classical logic is a crucial mathematical model of dispute or demonstration. So we should be interested in how we can characterize reasoning in this task in a way that it will bear some useful relation to reasoning outside this tiny domain, in say first-order classical logic, or even the much smaller, monadic first-order logic. This would be interesting. Tasks are not themselves interesting if there is no way of connecting them outside the laboratory or across domains. Small fragments are good for satisfying the exigencies of experiment, but they are of little interest in themselves. A good fragment generalizes—and for that one needs to know the goals (and norms) of the participant. There are also significant practical educational gains in understanding exactly why it is that participants have trouble differentiating the discourses of two logics. These problems are close to well known problems of mathematics education in distinguishing generation of examples from that of proofs (Stenning, 2002, chapter 5).

The real problem in this example is that there is more than one systematic reasoning goal that participants might adopt in doing the task as set—that is, more than one logic that might apply. The complaint quoted above is one clue here, though there are many others. The complaint is consistent with the idea that participants are adopting what might be called a “story understanding” task: roughly “What is the model of these premises which their author (presumably the experimenter) intends me to understand by them?” In nonmonotonic logics that capture this reasoning process, these are usually referred to as the *preferred* model (Shoham, 1987). This is cooperative nonmonotonic reasoning *to* a unique minimal model (i.e., *one* interpretation of the premises), as opposed to the adversarial monotonic reasoning from an interpretation, to conclusions true in *all possible models*, that classical logic specifies.

The proposal that cooperative communication worked through the contruction by speaker and hearer of what is now known as a “preferred model” appeared in Stenning (1975) and was condensed in Stenning (1978). Nonmontonic logic was new (McCarthy, 1980), and preferred models had to wait several more years (Shoham, 1987), but what was proposed informally was a direct route to cooperation for psychological process accounts (rather than an indirect Gricean pragmatics founded on adversarial classical logic). Stenning and Yule (1997) showed how subtle is the empirical discrimination of reasoning in classical logic and reasoning in nonmonotonic logic in the microcosms of the syllogism. The “Source-Founding Model” described there is a “shell” for capturing syllogistic reasoning processes, and it demonstrated that adopting a “guess the intended model” reasoning goal could actually yield all and only valid classical logical conclusions if the right model (roughly the “weakest”) was chosen, without any conceptual change to a new logic. The interesting psychological conceptual problems are about bald conceptual differences, but are actually difficult to resolve experimentally because the syllogism is so inexpressive. There is considerable evidence that most of the success participants achieve in syllogistic reasoning is achieved by preferred model construction. This is an example of the central importance of the empirical study of goals to the psychology of reasoning. Evans (2002) picks up the point about monotonic and nonmonotonic goals and about interpretation, but suggests no empirical approach other than variation in narrow instructions (rather than tasks) which Stenning and Yule (1997) showed to be inadequate.

**Figure 1:**
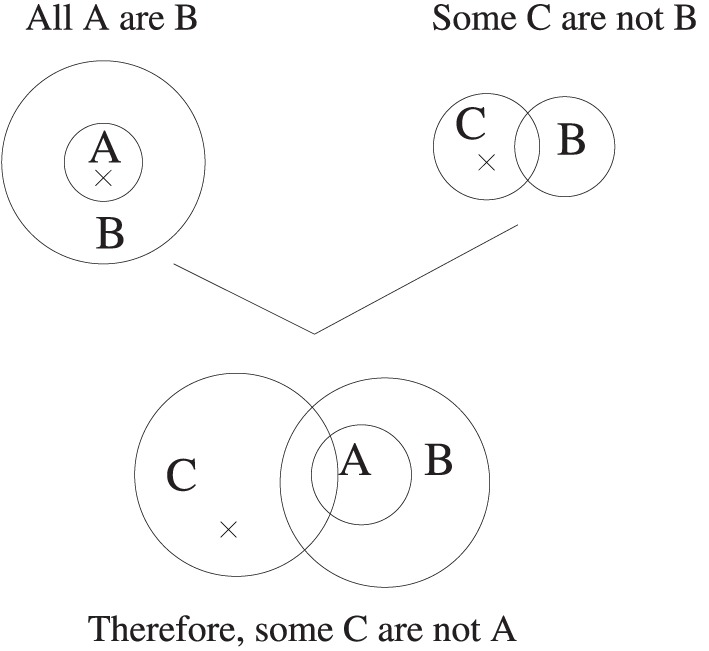
**Two premise diagrams unified in the Euler's Circles system of Stenning and Yule (1997)**. The crosses mark non-empty subregions. In the unified diagram, the A and C circles must be arranged to create the maximum number of minimal sub-regions compatible with the premises. In this case the A and C circles must intersect. Crosses whose minimal sub-region in the premise diagram have been bisected in this unification operation are deleted. Remaining crosses mark minimal models, and thereby indicate classically valid conclusions.

It is an immediate consequence that merely observing scores on the 64 syllogisms under different instructions in the conventional draw-a-conclusion task, will not tell us what logic a participant is reasoning with. We have to address the logical concepts that they have (for example, attitudes to conditionals with empty antecedents—more presently) and with them their processes of reasoning. We beg the reader's patience with some details which are important for understanding the role distinct goals (embodying distinct norms) play. We will use the diagrammatic methods this reference uses, though it also supplies analogous sentential ones. So for example, the syllogism *All A are B. Some C are not B* is represented by Figure 1.

In the final diagram, the single cross marks an element which is C but not A or B, which must exist in any model where the premises are true^9^. The choice of preferred models in the diagrams of each premise, combines with this construction of all consistent sub-regions, and with the rules for retaining or deleting the crosses, to ensure the result that any remaining cross represents an arbitrary individual with the properties defined by its subregion. The surprise is that this individual classically must exist if the premises are true. That is, the rules for choosing the nonmonotonically “preferred” model can conspire, in this tiny fragment of classical logic, to choose a model for the premises which has to exist in any situation where premises are true i.e., is a classically valid conclusion. This is of course not to say that participants who adopt a generally nonmonotonic goal for the task will automatically adopt the particular procedures required for getting classically valid preferred models: there are many parameterizations of the tweaking of nonmonotonic strategy. Informally, participants have to prefer the “weakest” model.

Stenning and Yule (1997) also provides a sentential algorithm which mirrors this graphical algorithm, as well as a “Source-Founding method” which is an abstract algorithm which captures what is in common between nonmontonic and classical methods. It shows the equivalence of the model manipulations in the diagrams with Aristotle's *ekthesis*. So it will be impossible to empirically distinguish participants' with classical norms from those with these “correctly tweaked” nonmonotonic reasoning norms by merely inspecting input premises and output conclusions. Yet identifying these norms is just what we argued psychology has to do to establish what implicit grasp of classical logic its participants have.

But help lies at hand. What has happened, in our nonmonotonic alternative method, to all those paradoxical properties of classical logic that bother every introductory logic student so much? For example, the paradoxes of material implication, whereby, from ¬*p* it follows that *p* → *q*; and from *q* it also follows that *p* → *q*. Or, for a related example, the conclusion that the King of France has been bald since the Revolution because there has been no King of France?: the problem of existential presuppositions. Besides, if the nonmonotonic tweaks get the classical answers, who needs to put up with these crises of classical logic?

So what is the psychological bottom line? The psychological half-way line, is that who needs classical logic is anyone who wants to go beyond the syllogism into the vastly more expressive first-order logic, and needs this still important model of demonstration and rational dispute (e.g., for mathematics, science, politics or the law). An experimenter might be tempted to the conclusion that this was just a bad fragment to pick, and progress to the psychological study of first-order or at least monadic first-order logic. There are formidable obstacles on that path, and no one has ventured down it far. But there is an alternative strategy within the syllogism. How can we get data richer than input-output pairings of premise-pairs and conclusions? If the conventional psychological task of presenting a pair of premises and asking whether any, and which of, the eight conclusions follows, brings forth nonmonotonic norms (albeit sometimes refined ones) from most participants, then perhaps what is needed is a new task and task context (dispute perhaps)? And what about getting participants to perform not just inferences, but also *demonstrations* of those inferences (by producing counterexamples)? This would provide evidence beyond input-output functions.

What are the quintessential features of classical reasoning that we should focus on in the data? The clues are in the paradoxes, though it requires some digging to unearth them. We are claiming, as is commonplace in traditional logical discussion, that classical logic is a model of dispute. What does this mean? Its concept of validity is that valid conclusions must be true in all models of the premises. What this means is that there must be no counterexamples (or “countermodels”). So classical logical demonstration is a doubly negative affair. One has to search for the *absence* of *counter*examples, and what is more, search exhaustively. A dispute starts from agreed and fixed premises, considers all situations in which these are all true, and wants to be certain that inference introduces no falsehood. The paradoxes of material implication immediately disappear. If *p* is false, then *p* ⊃ *q* cannot be false (its truth-table reveals that it can only be false if both *p* is true and *q* is false. (And truth tables is all there is to truth-functions). And the same if *q* is true. So given that *p* is false or *q* is true, we cannot introduce falsehood to true premises by concluding *q* from *p* ⊃ *q*. Everything follows from the nature of this kind of dispute, in which the premises must be isolated from other knowledge because they must be explicitly agreed, and in which no shifting of interpretation can be hidden in implications, or indeed in predicates. This latter is ensured by extensional and truth-functional interpretation. The “paradoxes” are thus seen as paradoxical only from the vantage point of nonmonotonic reasoning (our usual vantage point), whose norms of informativeness they violate. In dispute, proof and demonstration, the last thing one wants is the informativeness of new information smuggled in. And if you are engaged in telling a story, *failing* to introduce new information in each addition to the story will invoke incomprehension in your audience. Tautologies do little for the plot. This contrast is what we mean by each logic having its own discourse, and these two are incompatible.

Bucciarelli and Johnson-Laird (1999) earlier presented counterexample construction as an explicitly instructed task using syllogisms, though with a different partly graphical presentation of situations. Their purposes were to refute the claims of Polk and Newell (1995) that in the conventional draw-a-conclusion task, participants do not search for counterexamples, as mental models theory claimed that they understood that they *should*: ‘If people are unable to refute conclusions in this way, then Polk and Newell (1995) are certainly correct in arguing that refutations play little or no role in syllogistic reasoning’ (Bucciarelli and Johnson-Laird, 1999, page 270). Whilst their investigations of explicit countermodeling do, like ours, establish that participants can, when instructed, find countermodels above chance, they certainly do not counter Polk and Newell's claim that participants do not routinely do this in the conventional task on which mental models theory is based. Other evidence for Polk and Newell's skepticism now abounds (e.g., Newstead et al., 1999). But nowhere do any of these authors explicitly consider whether the participants' *goals* of reasoning in countermovement diverge from their *goals* of reasoning in the conventional task, even less whether they exemplify two different logics. At this stage, Mental Models theory was seen by its practitioners as the “fundamental human reasoning mechanism.” Another example of our dictum that it is exactly where homogeneity of reasoning is proposed, that normativism goes off the rails.

Searching for an absence of counterexamples then, is the primitive model-theoretic method of proof in the syllogism classically interpreted. The whole notion of a counterexample to be most natural, and best distinguished from an exception, needs a context of dispute. How do we stage one of those in the lab? Well, we tried the following (Achourioti and Stenning, in preparation). A nefarious character called Harry-the-Snake is at the fairground offering bets on syllogistic conclusions. You always have the choice of refusing the bets Harry offers, but if you think the conclusion he proposes does not follow from his premises (i.e., is invalid), then you should choose to bet against him. If you do so choose, then you must also construct a counterexample to his conclusion. Evidently we also have to explain to participants what we mean by a counterexample (a situation which makes both premises true and the conclusion false); what we mean by a situation (some entities specified as with or without each of the three properties A, B and C; and how to construct and record a counterexample. (In fact we use contentful material that does not affect likelihoods of truth of premises). Two features of this situation are that Harry-the-Snake is absolutely not to be trusted, and that it is adversarial—he is trying to empty your wallet. Another is that you, the participant, have chosen to dispute the claim Harry has made. You do not have to ask yourself “What if I thought this did not follow?” It has a vividness and a directness which may be important. Our selection of 32 syllogisms (unlike Bucciarelli and Johnson-Laird's) was designed to concentrate on the “no valid conclusion” problems which are at the core of understanding CL, and to allow analysis of the “mismatching” of positive and negative middle terms.

Our most general prediction was an increased accuracy at detecting non-valid conclusions. In the conventional task this is extremely low (37%): highly significantly worse than chance: in the new task it is 74%, significantly better than chance, and valid problems are 66% correct, which is also above chance. Valid problems are now harder, but the task now focusses the participant on the task intended. We also made some more specific predictions about a particular class of syllogisms which we call “mismatched,” in which the B-term is positive in one premise and negative (i.e., predicate-negated) in the other. Mismatching middle-term double-existential problems (e.g., *Some B are A, Some C are not-B*) “obviously” do not have single-element models, and so no valid conclusions. Compare a corresponding matched case *Some B are A, Some C are B* which yields as a unification model the single-element: (ABC). The most popular conclusion is *Some C are A*, drawn by 39% of participants. Note that this unification model is *not* a countermodel of this conclusion. With the mismatched example above, one cannot get a 1-element model. This difference between matched and mismatched double-existential problems and their most popular conclusions is systematic, as we describe below.

One might suppose that absence of valid conclusions is a general property of mismatching syllogisms because of the unification barrier to 1-element models, until one thinks about what happens if the first premise was instead *All B are A*. This universal premise would be satisfied by a single element model (such as *A not-B C*). But only if the negated B term is accepted as making the universal premise true by making its antecedent empty. That is, by the very same model which countermodels the existential case. Here is one place where the connection between CL's “paradoxes” and matching/mismatching shows up. Participants accepting the empty antecedent conditional as true can produce this one-element model.

So mismatching may serve as a tracer for issues with empty-antecedents. To find 1-element models for these mismatched problems requires accepting empty-antecedent conditionals as true. Now comes the question, do any of these syllogisms have valid conclusions? They can have 1-element models if one accepts empty antecedent conditionals, but are these models ones that establish valid conclusions? This model does not establish a valid conclusion anymore than the model (ABC) establishes a conclusion for *Some A are B. Some B are C*. In fact the problem does have a different valid conclusion *Some A are not C*. In summary, these mismatched problems provide a way to gain information about participants' intuitive grasp of empty-antecedent conditionals. And accepting empty-antecedent conditionals as true is a special case of accepting the paradoxes of material implication—the essential example of CL's “weirdness”—in the context of dispute. This is what we mean by looking for its “weirdnesses” as being the best evidence of implicit grasp of a logic. CL is weird in disputes; only from the non monotonic perspective, even for “logically naive” subjects.

If a participant has some implicit grasp of the one-element model generalization, and is happy with models satisfying conditionals by making their antecedent empty, then mismatched problems could behave differently than matched in this model-theoretic search-for-counterexample method: the striking logical feature (empty-antecedent conditionals being true) connects directly to an unexplored psychological feature. Mismatched problems, when we do the analysis, are actually observed to be slightly but significantly *harder* than matched ones in the conventional task of constructing a conclusion. To see how they might behave differently in countermodel search, one also needs to consider what the favorite conclusions are in the conventional task. For our example, the favorite response is *No C are A*. Now, we observe, that the model one gets by unifying the premises is (A not-B C) is immediately a *countermodel* of this popular conclusion (ie. some C *are* A in this model). If we take the matched and the mismatched problems in our experimental sample of 32, each paired with its favorite conclusion (from the meta-analysis), we find all the mismatched problems have this property that the unification model countermodels the favorite (and usually invalid) conclusions; whereas with the matched problems, the unification model is, in each case a *model* of the erroneous but favorite conclusion. This is evidently an empirical psychological generalization (favorite conclusions in a particular task have no logical status), though we clearly need the CL model-theory to even notice this piece of psychology. We predicted that *when looking for countermodels* (ie. doing CL), mismatched problems should be easier than mismatched ones.

What actually happens when Harry shows up? To cut a long story short, participants experience disputing with Harry-the-Snake as a much more arduous task than the conventional draw-a-conclusion task. They slow down by a factor of about three, an observation that already casts doubt on claims that this countermodel search takes place in the conventional task. Countermodel reasoning is hard work. Their overall accuracy of judgment of validity is not hugely increased, but it does not suffer from the extreme asymmetry of the conventional task. Both VC and NVC problems are done at a better than chance level. The control group in our conventional task control group are also much better than the literature average (these are highly selected students), but they are still asymmetrical in their success in the same way with VC easier than NVC problems. So we find the predicted improvement in detecting invalid conclusions, and we find that indeed whereas mismatched problems are somewhat harder than matched ones on the conventional task, they are substantially *easier* in countermodel reasoning in dispute with Harry, and that participants show evidence of accepting empty antecedent conditionals as true in the dispute task.

The pattern of errors in countermodel construction is consistent with a process by which participants first try to construct a premise model, then check to see if it is a countermodel, and if it is not, then adjust it to try to achieve a falsification of the conclusion. The problem appears to be that the adjustment often yields a model that falsifies the conclusion but is no longer a model of the premises. Mismatched models are more accurately countermodeled, and this is because the models that result from the unification of their premises are already countermodels of Harry's proposed conclusions, as illustrated above. This pattern that mismatched problems are actually easier for countermodel construction whereas they are harder in the conventional task strongly suggests that the majority of participants in the conventional task are operating proof-theoretically, probably by the nonmonotonic methods discussed above.

The countermodel construction data provides rich evidence that empty antecedent conditionals can be treated as true in this context. If the data is scored requiring existential presuppositions, most of the models produced for problems with one positive and one negative universal (i.e., no explicit existential premise) are not even models of the premises, let alone countermodels of the conclusion. A final observation that supports this general interpretation of a change of process invoked by dispute with Harry is that the orders of difficulty of problems in the conventional and in the Harry tasks are actually uncorrelated—an extremely strong result in support of the claim that here is the first task in the literature that produces substantial classical reasoning conducted on a classical conceptual basis. But even here, there are still many errors in countermodel reasoning. The usual justification of the conventional task is that the order of the difficulty of problems is systematic and always the same. The first time anyone makes a comparison with a context designed to invoke a different logic, one finds this order of difficulty changes radically.

Clarifying the intended goals of reasoning (norms to adopt) for participants is one of the few ways we have of pursuing the question whether there are contexts in which participants intuitively understand the concepts of a logic. One can imagine the objection that we have told them to do countermodel reasoning and so it is not surprising that they appear to reason classically. But this is a psychologically bizarre idea. It's no use telling these participants to reason in classical logic because they do not explicitly know what that means. They do have some grasp of what a dispute is, and the role of counterexamples therein—the discourse of dispute. We are merely negotiating a common reasoning norm with our participants. If they did not understand these things, the negotiation would not succeed. We doubt it succeeds with all our participants. But we certainly do not instruct them about what to do with empty antecedent conditionals. And sure enough, we see the peculiarities of classical logical reasoning in their performance. This is just what the psychological foundations of classical logic are: an inexplicit intuitive grasp of dispute. These empirical conceptual questions such as “What do participants ‘know’ about classical logic?” have far more psychological reach than questions about how many syllogisms do participants get “right” in any particular contextualized task where the goals are not understood the same way by participant and experimenter, or across participants.

Participants are, unsurprisingly, not tactically expert. But here at least is the beginning of an empirical program to study this kind of reasoning in contradistinction to various kinds of nonmonotonic reasoning. Although the two may overlap within the syllogism, outside the syllogism they diverge. And even within the syllogism, here is evidence that the two very different reasoning goals are operative in different contexts, and lead to radically different mental processes, each incomprehensible without an understanding of the different logical goals, and of the participants' informal contextual understandings of their logical goals.

## 4. Reasoners' goals in the new probabilistic paradigm

Classical logic has been found wanting as a complete model of human inference for many reasons, some of which we have already covered. The “new paradigm” of subjective probabilities aspires to become its replacement (Over, 2009; Oaksford and Chater, 2013). A central question has been whether people's interpretation of indicative conditionals, ‘if *A*, then *B*’, is given by the material conditional *A*⊃ *B* (see Table 1 for a reminder of its truth values) or the conditional probability *P*(*B*|*A*). There is evidence that in some circumstances participants do indeed reason that the probability of ‘if *A*, then *B*’ is given by *P*(*B*|*A*), both when dependencies between antecedent and consequent are expressed in the task through joint frequencies about patterned cards (Evans et al., 2003; Oberauer and Wilhelm, 2003) and when dependencies are derived from causal beliefs (Over et al., 2007). These interpretations also extend to conditional bets such as “I bet you 1 Euro that if the chip is square then it is black” (Politzer et al., 2010), a result which is predicted by foundational work on subjective probability by Bruno de Finetti (Milne, 1997, gives an overview).

**Table 1:** **Truth values of the classical logic material conditional (*A* ⊃ *B*), conjunction (*A* ∧ *B*), and semantic values of the conditional event (*B*|*A*) and biconditional event (*B*|*A*) ∧ (*A*|*B*), where 1 denotes “true,” 0 denotes “false,” and *u* denotes “undefined”**.

***A***	***B***	***A* ⊃ *B***	***A* ∧ *B***	***B*|*A***	**(*B*|*A*) ∧ (*A*|*B*)**
1	1	1	1	1	1
1	0	0	0	0	0
0	1	1	0	*u*	0
0	0	1	0	*u*	*u*

The conditional event, *B*|*A*, is often defined only for conditional probabilities in terms of the ratio formula,

$$P(B|A)=\frac{P(A\wedge B)}{P(A)}$$

under the condition that *P*(*A*) > 0. Coherence-based probability logic (CPL), proposed as a competence model for how people reason (Pfeifer and Kleiter, 2009), makes this a primitive, *B*|*A*, which is “undefined,” “void,” or “undetermined” when the antecedent is false, matching how participants often interpret the conditional when reasoning under certainty (Johnson-Laird and Tagart, 1969). Although this interpretation is often called the “defective” conditional, there is a long history of justification suggesting that there is nothing defective about it. CPL derives a semantics for conditional probabilities, providing a bridge between certainty and uncertainty. This explains why people who use a “defective” conditional when reasoning about certainty also reason using conditional probabilities for uncertain conditionals (Evans et al., 2007, show this empirical link): it's the same underlying conditional.

Hailperin (1996) provides a further analysis of this conditional event (he calls it the “suppositional”) in terms of a more primitive operator in an extension of classical propositional logic, “don't care” logic. We present this in a some detail here as it shows clearly the relationship with classical logic. Let 1 denote “true,” 0 denote “false,” and *u* denote “undefined.” The ordering on these semantic values is 0 ≤ *u* ≤ 1. This leads to natural *min* and *max* functions for deciding the minimum and maximum of two values which are used to define conjunction and disjunction, respectively. Let *min*(*x, y*) = *z*; then *z* is either the *x* or *y* and chosen such that *z* ≤ *x* and *z* ≤ *y*, i.e., the value is less than or equal to both *x* and *y* according to the ordering above. Let *max*(*x, y*) = *z*; then again *z* is one of the *x* or *y* and *z* ≥ *x* and *z* ≥ *y*, i.e., the value is greater than or equal to both *x* and *y*. Some examples to illustrate: *max*(0, 1) = 1 and *min*(0, 1) = 0. If *x* and *y* are the same value then the answer is that value for both *min* and *max*. When the *u* value is included then *max*(0, *u*) = *u* (since *u* is greater than or equal to both 0 and itself) and *min*(0, *u*) = 0 (since 0 is less than or equal to both *u* and itself). Finally 1 − *u* = *u* (this is used for defining negation). *U* is a semantic function from formulas to semantic values, i.e., one of 0, 1, or *u*, as follows:

$$\begin{array}{l} \;U(\neg A)=1-U(A)\\ U(A\wedge B)=min(U(A),U(B))\\ U(A\vee B)=max(U(A),U(B)) \end{array}$$

If we use only 1s and 0s, this is also the semantics of classical propositional logic. When *u* is included, then the semantics is equivalent to Kleene's strong 3-valued logic (Kleene, 1952) which turns out to be useful for the semantics for logic programming (Fitting, 1985). Hailperin (1996) introduces an additional “don't care” unary connective, △, with a semantic value defined:

$$U(△A)=\{\begin{array}{ll} 0, & \text{if }U(A)=0\\ u, & \text{otherwise} \end{array}$$

If *A* is true then △*A* evaluates to *u*; otherwise it has the same semantic value as *A*. This allows *B*|*A* to be defined △ ¬*A* ∨ (*A* ∧ *B*), giving the same semantic values as CPL. Note the similarity with the disjunctive expression of the material conditional, ¬*A* ∨ *B*, which is equivalent to ¬*A* ∨ (*A* ∧ *B*). Both have the same semantic value when the antecedent is true, equivalent to a conjunction. The disjunct highlights the difference when the antecedent is false: for the conditional event the conditional is undefined but for the material conditional it is true. (This is one of many non-classical truth semantics; Baratgin et al. (2013) provide other interesting examples of further logical components which are useful for psychological theorizing.) Individuals and quantifiers are missing from this semantics, which limits its ability to model discourse; for instance it is not clear how to model an interpretation of “most logicians who develop a logic love it.”

Returning to the psychology, there are interesting twists to the new paradigm story. It turns out that the experimental data also require us to model a defective biconditional, what Fugard et al. (2011b) named the biconditional event. This is expressed as (*B*|*A*) ∧ (*A*|*B*) (see Table 1 for its semantics values) and is equivalent to *A* ∧ *B*|*A* ∨ *B*. Developmental studies show that 12 year olds respond mostly with conjunctions, then by age 16 biconditional event interpretations appear before disappearing again in adults (Gauffroy and Barrouillet, 2009). In adults, it is well replicated that nearly half of participants interpret the conditional as a conjunction, *A* ∧ *B*. Shifts of interpretation have also been found within adults: many participants who begin with a conjunction interpretation change that interpretation (without feedback) to a conditional probability (Fugard et al., 2011b; Pfeifer, 2013). Participants occasionally are explicit about this, describing their reasoning about what they think they are supposed to do and changing their goals, occasionally swearing as they do so, a sure sign of norms awry.

Gauffroy and Barrouillet (2009) explain the developmental trend in a revision of mental models theory. Essentially the idea is that more slots of memory are required as one moves from conjunction—produced by heuristic processes immune to strong developmental changes' (p. 274)—through biconditional event, to conditional event. All reasoners are assumed to have the same reasoning goals, they just fail if they have insufficient memory. Fugard et al. (2011b) instead argued that there are two main stages to reasoning about these sorts of conditionals when the dependencies are expressed in the stimulus, for instance as colored cards. First one has to visually perceive the dependencies, which requires attending to all cases. If you are reasoning about new evidence then you first have to examine the evidence. All evidence is initially relevant, even those cases where the antecedent is false, as you can only tell it is false once you have seen it. The developmental trend can be seen as strategic ignorance when all the evidence has been examined: first from no narrowing of hypothetical scope for conjunctions (*A* ∧ *B*), to focusing on only those cases where either antecedent or consequent are true (*A* ∧ *B*|*A* ∨ *B*), finally to only those cases where the consequent is true, (*A* ∧ *B*|*A*) which is equivalent to the conditional event *B*|*A*. Further support for this model is that conjunctions seem to disappear in Experiment 1 by Over et al. (2007) where instead of reading dependencies from the stimulus, they were taken from beliefs, e.g., that “If nurses” salaries are improved then the recruitment of nurses will increase. There is no need to consider evidence when you are asked your opinion. This hypothetical narrowing could be for many reasons. Perhaps there are variations in pragmatic language function which affect the interpretation of what the experimenter wants. Another explanation is that working memory and reasoning processes have competing goals: represent everything that one sees versus reason about top-down goals concerning the present task (Gray et al., 2003). The two could well be related and influence reasoning about goals. People can switch goals for resource reasons.

The “new paradigm” is often presented as providing the semantics for the conditional as illustrated by ‘the Equation’: *P*(‘if *A*, then *B*’) = *P*(*B*|*A*). But interpretation is required for probabilities too. Fugard et al. (2011a) showed that a relevance pragmatic language effect, well replicated for non-probability problems in the classical logic paradigm, also affects probabilistic theories of conditionals. Consider the following sentence about a card.

If the card shows a 2, then the card shows a 2 or a 4.

In the old binary paradigm, people tend to think this sentence is false (though with the usual individual differences) since the possibility that the card could be a 4 seems irrelevant if you know it is a 2. Fugard et al. (2011a) found that when participants were shown four cards, numbered 1 to 4, and told that one has been chosen at random, many thought the probability of this sentence is 0. Probability logic (with the simple substitution interpretation) predicts that they would say the probability is 1. Given the same cards but instead the sentence

If the card shows a 2, then the card shows an even number,

most participants give the probability 1 which is now consistent with the Equation. The new paradigm of transforming ‘if’s into conditional events does not predict this different in interpretation. Here, as for much of the psychology of reasoning, there are differences between participants in interpretation and not all reasoners have the goal to take relevance into consideration. Fugard et al. (2011a) found no association between irrelevance aversion and tendency to reason to a conjunction probability, suggesting that the two processes are logically and psychologically distinct.

The problem for the probability story, as the semantics above shows, is that the disjunction in probability logic is the same as the disjunction in classical logic, so this provides a clue for a solution. Schurz (1991) provided an extension of classical logic for interpretations like these: sentence φ is a *relevant* conclusion from premises Γ if (a) it follows according to classical logic, i.e., Γ ⊢ φ holds, and (b) it is possible to replace any of the predicates in φ with another such that φ no longer follows. Otherwise φ is an irrelevant conclusion. Take for instance the inference *x* = 2 ⊢ *x* = 2 ∨ *x* = 4. Since *x* = 4 can be replaced with any other predicate (e.g., for the synesthetes *red*(*x*)) without affecting validity, the conclusion is irrelevant. However for the inference *x* = 2 ⊢ *even*(*x*), not all replacements preserve validity, for instance *odd*(*x*) would not, so the conclusion is relevant. Fugard et al. (2011a) propose adding this to the probability semantics.

Reasoners still have goals when they are reasoning about uncertain information. There are competing processes related to working memory and planning, which could explain developmental processes and shifts of interpretation within participants. Goals related to pragmatic language, such as relevance, are also involved in uncertain reasoning. The investigations above highlight the importance of a rich lattice of related logical frameworks. The problems of classical logic have not gone away since, as we have shown, much of classical logic remains in the 3-valued semantics. Rather than only examining whether or not support is found for the probability thesis, instead different norms are needed through which to view the data and explain individual differences. These norms need to bridge back to the overarching goals reasoners have.

We finish this section with a comment on the treatment of this same problem by Bayesian modeling. The probability heuristic model (PHM) of Chater and Oaksford (1999) was one of the first to protest against the idea that classical logic provided the only interpretation of syllogistic performance. A protest with which we evidently agree. This Bayesian model certainly changes the measures of participants accuracy in the task. For the present argument, two observations are relevant. Firstly, PHM is probably best interpreted as a probability-based heuristic *theorem prover* for classical logic. The underlying logic is still in classical logic and even includes first-order logic statements. The truth of the propositions is assessed classically. This means that despite the rejection of the formal model of classical logic, it has not departed very far. PHM does not propose an alternative interpretation of the goal of reasoning as we do here. Secondly, once the Bayesian model is in place, the psychology stops. There is no motivation for seeking other models of other qualitatively different kinds of reasoning, because probability based models are supposed to account for all reasoning. This may be a consequence of at least a whiff of poor prescriptivism here, and bears out the claim we made that this problem is found wherever one framework is seen as sufficient. In contrast, in a multiple-logics approach, contrast between logics is a rich source of insight and guidance as to how to find the relevant psychological evidence. It should be evident from this example that logic can make empirical experimental analysis much richer. Instead of hundreds of experiments on essentially the same design, one gets a vista of empirical questions to explore.

## 5. Conclusions

A variety of formal systems, with their different constitutive norms, and their different consequences for the regulative norms of their users, will be required for modeling the different goals of human reasoning. The main goal of the experimental program of psychology of reasoning and decision at this point should be to find contexts in which participants will exhibit their maximum grasp of each system. Exploration can then spread out to investigate how the logics work together in more complex tasks; how participants can generalist from these focal points; and how teaching affects what they can do. If we win our bet on Harry as a good teacher of an explicit grasp of the logical differences between disputes and stories, and we can show the rudiments of classical logic in a good proportion of participants' performances, then that does not mean that CL “won” over nonmonotonic logics such as LP, or over probability logics, or whatever other logics can be shown to have their contexts. It means we know a little more about where to look for classical logic's psychological roots. We can ask how do these cognitive foundations develop, and what individual and social experiences affect them. We can ask how people at different stages of development and education experience the phenomenology of their reasoning. We can ask how best to achieve educational goals of making explicit students' knowledge of logics. And so on.

In many cases, the empirical discriminations between logics are surprisingly hard. Natural languages often do not provide adequate (or indeed any) cues to intended reasoning goals. People are good at recognizing the goals in customary rich social contexts (few mistake a dispute for a story), but the lab removes all these cues, as do many real-world professional contexts. Much effort is currently going onto the issue of what probability theory is good for, but little into where nonmonotonic logics are to be preferred. Deep knowledge of the logical and computational properties of these systems is available outside psychology but often shunned. Formal systems such as logics and probability are still conventionally seen as competing with psychology for explanations of reasoning. A recent prominent example of this attitude (here to probability rather than logic) is Jones and Love (2011).

Bayesian modeling of cognition has undergone a recent rise in prominence, due largely to mathematical advances in specifying and deriving predictions from complex probabilistic models. Much of this research aims to demonstrate that cognitive behavior can be explained from rational principles alone, without recourse to psychological or neurological processes and representations.

Bayesians would dispute whether they claim to explain in rational terms *alone*. We would disagree with many of their “rational explanations.” One might certainly feel disappointed if rational explanations were all of psychology. One of the reasons for our detailed examples is to show that logical bases for explanations do not mean they cannot reveal psychological processes. A huge amount of research in a descriptivist style has failed to make the most important empirical distinctions about which interpretations of the tasks are adopted. But having said all this, to challenge the idea that rational explanations are part of psychology is truly extraordinary. What is needed is more attention to norms, and to the way the constitutive norms of formal systems give rise to regulative norms for their use, and above all, on participants' access to these norms of both kinds.

There is no alternative to a psychology of reasoning which has a rich theoretical vocabulary of reasoning norms, which constitute different goals, and a fine nose for finding the contexts of reasoning that call for the goals, based on the norms of the logical models. Descriptivism never worked in any science.

### Conflict of interest statement

The authors declare that the research was conducted in the absence of any commercial or financial relationships that could be construed as a potential conflict of interest.

## Footnotes

^1^For example, one of the prominent accounts of long-term/working-memory interactions (Anderson, 1983) contains a production system which is a specific implementation of LP, the nonmonotonic logic we employ here. So logic is also not so distant from the WM component of bounded rationality. Many psychologists regard retrieval of relevant information from long-term memory as memory rather than reasoning. It is certainly memory, but equally certainly reasoning.

^2^For what Elqayam and Evans (2011) argue against, the term “normativism” seems to us more appropriate than “normativity.” This is indeed the term that these authors use, while many of the commentators talk about “normativity.” This is not to say that the differences of opinion are merely terminological; it is rather the choice of key terms that is influenced by the theoretical positions adopted.

^3^The authors seem to take issue with the concept of “error” because it evidences the use of norms: ‘While the term “normative” has been dropped, the term “error” has not: A recent book (Stanovich, 2009) presents an extensive discussion of the source of reasoning and decision-making errors, implying norms’. (Elqayam and Evans, 2011), p.242

^4^We discuss constitutive and regulative norms and their relations also in Achourioti et al. (2011).

^5^It must be clear by now that we do not subscribe to a distinction of formal systems into normative and descriptive; it is rather the use we put these systems to in accounting for human reasoning that can be labeled as such.

^6^Logicians produce “embedding theorems” which prove that one logic can be “embedded” within another, often when the two look rather incompatible. It does not follow that the more encompassing logic is an appropriate cognitive model for the encompassed systems' cognitive applications.

^7^These “glitches” turn out to be at the heart of some of the psychological issues about CL: more below.

^8^Percentage responses here and following are taken from the metanalysis by Khemlani and Johnson-Laird (2012).

^9^The diagrammatic system is described in more detail in the reference above and also in Stenning and Oberlander (1995), e.g., Figure 2. In the variant used here, existential presuppositions are made for universals, because that assumption is commonplace in the psychology literature. Below we see that it is not clearly the right assumption when the task context changes to dispute.
